# A Very Rare Case of Megalencephalic Leukoencephalopathy With Subcortical Cysts in a Child Born of Non-Consanguineous Marriage in a Non-Predisposed Community

**DOI:** 10.7759/cureus.16941

**Published:** 2021-08-06

**Authors:** Harneet S Randhawa, Jasneet Randhawa, Anagha Kulkarni, Akshay More, Akshay Jain

**Affiliations:** 1 Department of Radiology, Government Medical College Baramati, Pune, IND; 2 Department of Resident Medical Services, Fortis Escorts Hospital, Amritsar, IND; 3 Department of Pediatrics, Lokmanya Tilak Municipal General (LTMG) Hospital, Mumbai, IND; 4 Department of Interventional Radiology, Lokmanya Tilak Municipal General (LTMG) Hospital, Mumbai, IND; 5 Department of Radiology, Government Medical College, Kolhapur, IND

**Keywords:** mlc, von der knaap, megalencephalic leukoencephalopathy, subcortical cysts, rare autosomal recessive disorder, consanguinity, ataxia, seizure, temporal cysts, white matter changes on mri

## Abstract

Megalencephalic leukoencephalopathy (MLC) with subcortical cysts is a very rare white matter disorder characterized predominantly by motor developmental delay and seizures in a child with macrocephaly. Extrapyramidal symptoms, ataxia and mental retardation may also occur. Only a few cases of the disease have been reported worldwide with most of them showing an autosomal recessive pattern of inheritance. In India, most cases have been reported in Agrawal community. Here, we present an interesting case of MLC in a child born in non-Agrawal community to a non-consanguineous marriage. By reporting this case we intend to increase the research horizon and increase the published literature for atypical cases of MLC.

## Introduction

The credit for identifying MLC goes to Dutch pediatric neurologist Marjo S Van der Knaap and hence, the disease is often referred to as Van der Knaap disease. In 1995, she studied brain imaging in children with macrocenphaly and leukoencephalopathy who presented with motor developmental delay, seizure and other symptoms like ataxia. She described the imaging characteristics of the disease as peripheral and subcortical white matter involvement which is diffuse, homogenous and supratentorial with sparing of grey matter and central white matter. Subcortical cysts involving bilateral anterior temporal region and sometimes frontoparietal region are also noted [1]. Since the discovery of the disease, only a few cases (over 150) have been reported worldwide [2]. Even though exact prevalence is not known; Orphanet mentions a prevalence of < 1/1,000,000 [3]. In the Indian population, the cases have been reported in Agrawal community and the disease is sometimes referred to as Agrawal disease [4].

MLC is mostly inherited as an autosomal recessive disorder (MLC1 and MLC2A), and a small proportion as autosomal dominant (MLC2B). Underlying defects are noted in MLC1 and HEPACAM genes, thus affecting the astrocytes at blood-brain barrier causing abnormal extracellular potassium dynamics, swelling and vacuolation of white matter [5-7]. Phenotypically it is divided into two variants; the Classic phenotype (MLC1 and MLC2A) and an Improving phenotype (MLC2B). Improving phenotype is a milder form where motor functions improve after infancy. The main clinical features include macrocephaly, mild developmental delay (predominantly gross motor domain), seizures that are often controlled with medicine, extrapyramidal symptoms, some level of intellectual disability and behavioral abnormalities [6]. MLC is diagnosed based on clinical and imaging features, genetic studies if available can be helpful. Correct diagnosis is important for proper counselling of parents, genetic counselling to avoid future recurrence and better management of disease. No definitive treatment is available for the disease and so physical therapy along with anti-epileptics for seizures are used for management [6].

## Case presentation

**Figure 1:**
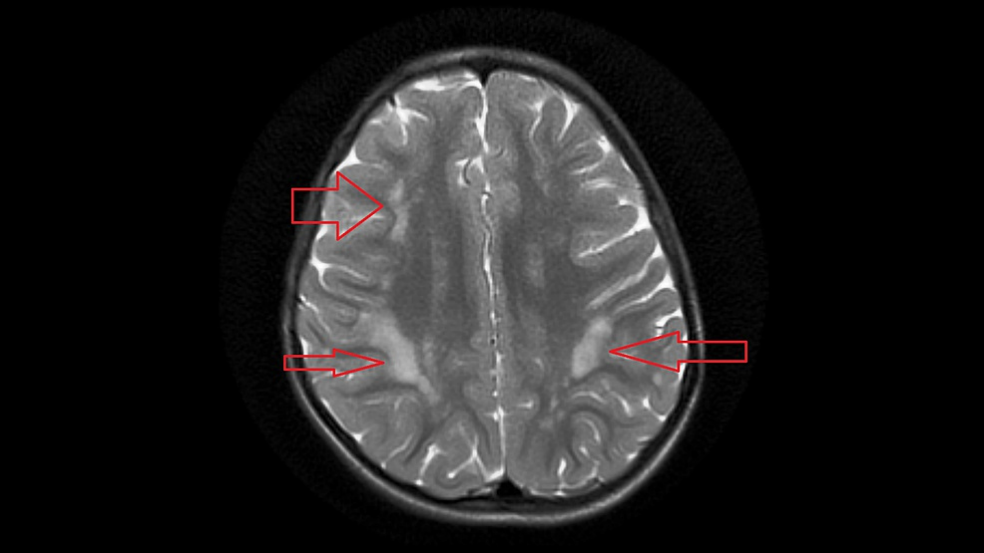
Axial T2W image showing discrete and confluent hyperintensities involving bilateral subcortical white matter in frontoparietal region.

**Figure 2:**
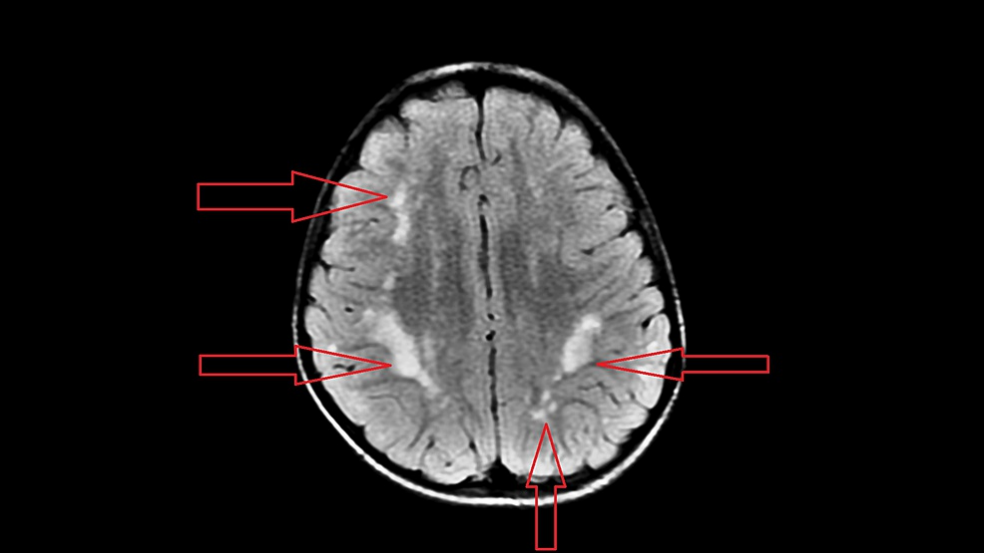
Axial T2 FLAIR image corresponding to T2W image in Figure 1. FLAIR: fluid-attenuated inversion recovery.

**Figure 3:**
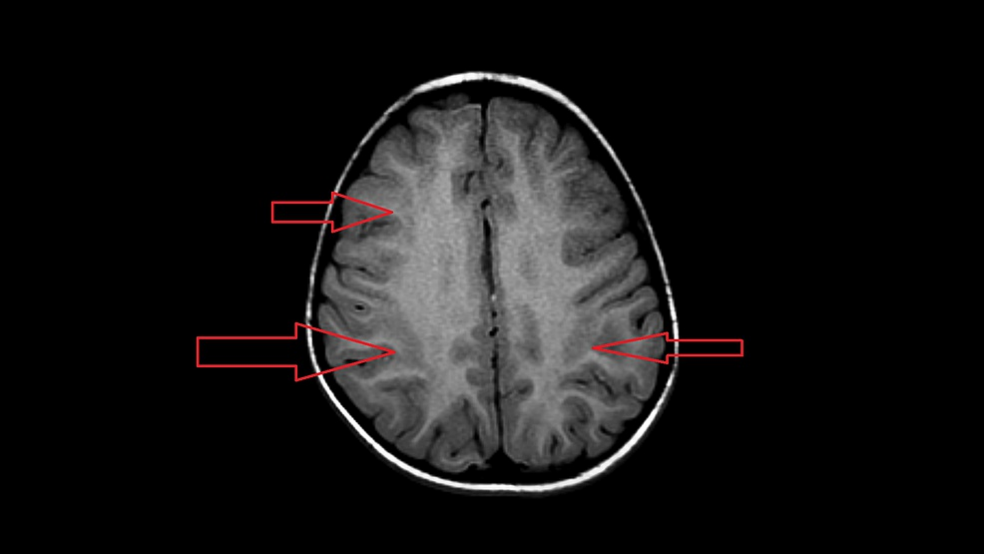
Axial T1W image showing hypointensities corresponding to abnormal T2 signal in Figure 1.

We present a case of a three-year-old female child born to parents from a non-Agrawal community, who had a non-consanguineous marriage. The child had macrocephaly and later developed seizures during infancy which were controlled with medication. Developmental milestones were delayed; as she started walking with support around 15 months and without support only after two years, she still had difficulty running or walking on stairs after three years. Mild lag was also present in social and language domains; she was unable to construct phrases or sentences and had a vocabulary of 10-15 words. Birth history was normal; she was born by normal vaginal delivery at term and there was no history of birth asphyxia. Family history did not suggest any similar features in close relatives. The general physical examination was normal. Routine blood profile and electrolytes were normal. Patient was clinically diagnosed with global developmental delay with seizures and was referred to our radiology department for MRI brain. MRI showed multiple ill-defined discrete and confluent T2 hyperintensities in subcortical and peripheral white matter with corresponding hypointensities on T1W images and no inversion on fluid-attenuated inversion recovery (FLAIR) (Figures 1, 2, 3).

**Figure 4:**
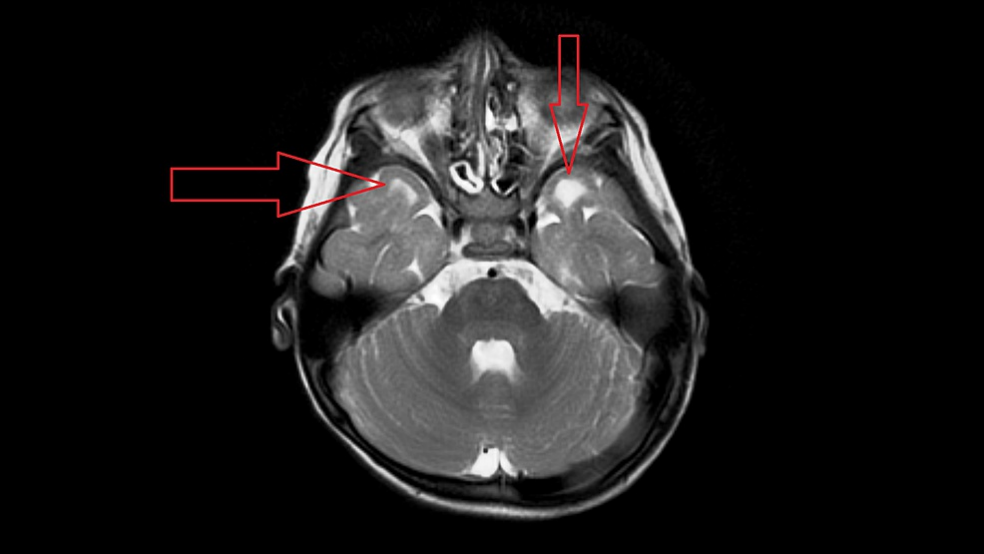
Axial T2W image showing subcortical cystic areas in bilateral anterior temporal lobes.

**Figure 5:**
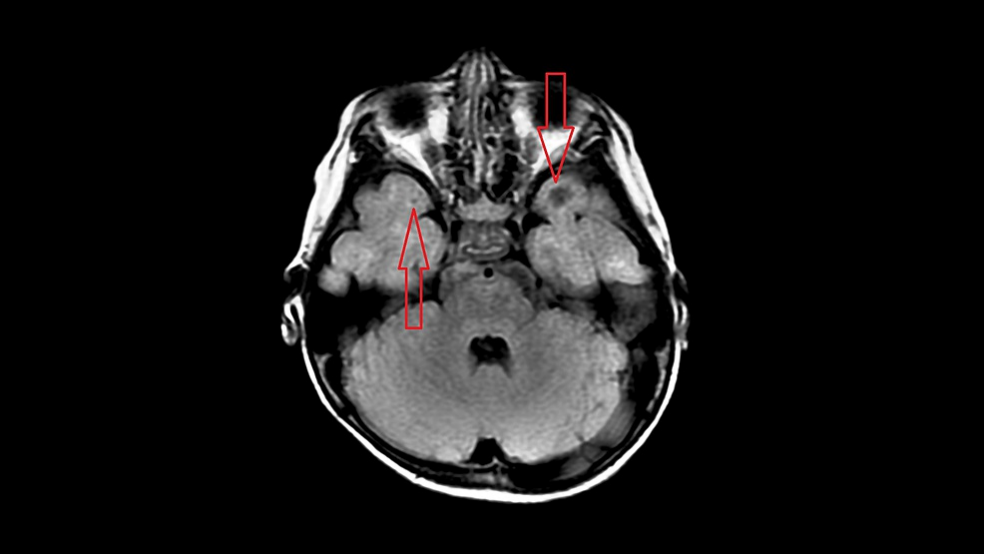
Axial T2 FLAIR image corresponding to T2W image of Figure 4 showing inversion of cystic fluid. FLAIR: fluid-attenuated inversion recovery.

No diffusion restriction or blood products were noted on DWI and GRE respectively. No abnormal post-contrast enhancement was noted. Subcortical cystic change was noted in bilateral anterior temporal region (Figures 4, 5).

**Figure 6:**
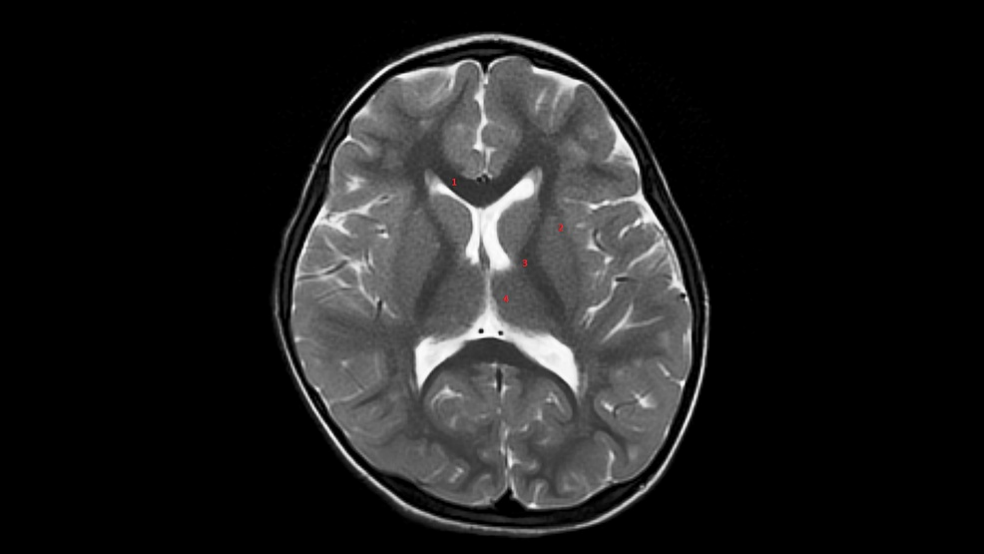
Axial T2W image showing normal basal ganglia and normal corpus callosum (1), unaffected external capsule (2), normal Internal capsule (3) and spared thalami (4).

No abnormal NAA peak was noted on spectroscopy. Deep white matter and basal ganglia were normal (Figure 6).

Combining the clinical profile with typical imaging findings the diagnosis of megalencephalic leukoencephalopathy with subcortical cysts was made. Due to non-availability of any specific treatment, parents were properly counselled. Physical and speech therapy was advised and antiepileptic treatment was continued.

## Discussion

Here we report an atypical case of a very rare disease. MLC is mostly inherited as an autosomal recessive disorder in predisposed populations i.e. Turks, Jews and Agrawal community of India [6]. Parents of our patient had a non-consanguineous marriage and are from a non-Agarwal community. There was no history of a similar condition in close relatives. Less than a handful of such cases have been reported worldwide. One such case was reported by Roy et al. in 2015 [8].

Various etiologies result in developmental delay and seizures, such as neurovascular insult (Hypoxic ischemic encephalopathy), Congenital/structural brain abnormalities, metabolic and white matter diseases, genetic syndromes and other non-specific causes [9,10]. Absence of history suggestive of hypoxic insult along with sparing of cortex, periventricular region, basal ganglia and deep white matter on MRI ruled out hypoxic etiology [11]. Structural brain abnormalities that cause developmental delay and seizures often affect the cortex. These include focal cortical dysplasia, lissencephaly, heterotropias, polymicrogyria etc [12]. No such changes were noted on our patient's imaging. Numerous white matter disorders result in developmental delay and seizures. However, differentials for macrocephaly with diffuse leukoencephalopathy are few and include Alexander disease, Canavan disease, GM1/GM2 ganglisidosis, l-2-hydroxyglutaric acid aciduria [6]. Absence of basal ganglia and thalami involvement, no NAA peak on spectroscopy and cysts in anterior temporal lobe ruled out Canavan disease. Lack of enhancement on post contrast images and no frontal predominance was not consistent with Alexander disease. Sparing of dentate nucleus, basal ganglia and thalami ruled out l-2-hydroxyglutaric acid aciduria and gangliosidosis [6]. Considering the characteristic imaging findings of megalencephalic leukoencephalopathy with subcortical cysts as described by M S Van der Knaap and ruling out other differentials as described above the final diagnosis of MLC was reached. Parents of the child were properly counselled about the course and prognosis of the disease. Physical therapy, speech therapy and special schooling was advised. Since most cases of MLC are inherited as autosomal disorders a detailed genetic counselling was advised.

## Conclusions

In conclusion, atypical cases of megalencephalic leukoencephalopathy with subcortical cysts are extremely rare and only a few have been reported in the literature. By reporting this case we are trying to increase the knowledge about the same. MLC should always be considered in patients having typical imaging pattern, even when there is no significant family history and diagnosis should be reached after all its close differentials are ruled-out. Most reported typical cases have autosomal recessive inheritance in predisposed communities, and not enough genetic research has been done in atypical cases. Future genetic studies are needed to further help clear the pattern of inheritance in atypical cases and a better understanding of disease.

## References

[1] van der Knaap MS, Barth PG, Stroink H, van Nieuwenhuizen O, Arts WF, Hoogenraad F, Valk J (1995). Leukoencephalopathy with swelling and a discrepantly mild clinical course in eight children. Ann Neurol.

[2] Jhancy M, Al Homsi A, Chowdhury F, Hossain S, Ahamed R (2020). Van der Knaap disease (vanishing white matter) - unusual presentation in a neonate: a case report. Neurol India.

[3] (2020). Rare diseases are rare, but rare disease patients are numerous. https://www.orpha.net/consor/cgi-bin/index.php.

[4] Singhal BS, Gursahani RD, Udani VP, Biniwale AA (1996). Megalencephalic leukodystrophy in an Asian Indian ethnic group. Pediatr Neurol.

[5] Estévez R, Elorza-Vidal X, Gaitán-Peñas H (2018). Megalencephalic leukoencephalopathy with subcortical cysts: A personal biochemical retrospective. Eur J Med Genet.

[6] van der Knaap MS, Abbink TEM, Min R (2003). Megalencephalic Leukoencephalopathy with Subcortical Cysts. 2003 Aug 11 [updated 2018 Mar 29].

[7] Dubey M, Brouwers E, Hamilton EM (2018). Seizures and disturbed brain potassium dynamics in the leukodystrophy megalencephalic leukoencephalopathy with subcortical cysts. Ann Neurol.

[8] Roy U, Joshi B, Ganguly G (2015). Van der Knaap disease: a rare disease with atypical features. BMJ Case Rep.

[9] Ali AS, Syed NP, Murthy GS, Nori M, Abkari A, Pooja BK, Venkateswarlu J (2015). Magnetic resonance imaging (MRI) evaluation of developmental delay in pediatric patients. J Clin Diagn Res.

[10] Momen AA, Jelodar G, Dehdashti H (2011). Brain magnetic resonance imaging findings in developmentally delayed children. Int J Pediatr.

[11] Chao CP, Zaleski CG, Patton AC (2006). Neonatal hypoxic-ischemic encephalopathy: multimodality imaging findings. Radiographics.

[12] Bozzi Y, Casarosa S, Caleo M (2012). Epilepsy as a neurodevelopmental disorder. Front Psychiatry.

